# Efficacy of Simparica Trio™, a novel chewable tablet containing sarolaner, moxidectin and pyrantel, against induced hookworm infections in dogs

**DOI:** 10.1186/s13071-020-3951-4

**Published:** 2020-03-01

**Authors:** Csilla Becskei, Mirjan Thys, Kristina Kryda, Leon Meyer, Susanna Martorell, Thomas Geurden, Leentje Dreesen, Tiago Fernandes, Sean P. Mahabir

**Affiliations:** 1 Zoetis, Veterinary Medicine Research and Development, Mercuriusstraat 20, 1930 Zaventem, Belgium; 2 Zoetis, Veterinary Medicine Research and Development, 333 Portage St., Kalamazoo, MI 49007 USA; 3 ClinVet International, Uitsigweg, Bainsvlei, 9338 Bloemfontein, Republic of South Africa; 4 ClinVet International, Douar Dbabej, Beni Yekhlef, 28815 Mohammedia, Morocco; 5 Zoetis, Manufacturing & Research, Carr. de Camprodon, s/n, La Vall de Bianya, 17813 Girona, Spain

**Keywords:** Ancylostomatids, *Ancylostoma caninum*, *Uncinaria stenocephala*, Immature stages, L_4_ larvae, L_5_ immature adults

## Abstract

**Background:**

Ancylostomatids (‘hookworms’) are among the most important zoonotic nematode parasites infecting dogs worldwide. *Ancylostoma caninum* and *Uncinaria stenocephala* are two of the most common hookworm species that infect dogs. Both immature and adult stages of hookworms are voracious blood feeders and can cause death in young dogs before infection can be detected by routine fecal examination. Hence, treatment of both immature and adult stages of hookworms will decrease the risk of important clinical disease in the dog as well as the environmental contamination caused by egg-laying adults, which should reduce the risk of infection for both dogs and humans. The studies presented here were conducted to evaluate the efficacy of a novel, oral chewable tablet containing sarolaner, moxidectin and pyrantel (Simparica Trio™), against induced larval (L_4_), immature adult (L_5_) and adult *A. caninum*, and adult *U. stenocephala* infections in dogs.

**Methods:**

Eight negative-controlled, masked, randomized laboratory studies were conducted. Two separate studies were conducted against each of the target parasites and stages. Sixteen or 18 purpose bred dogs, 8 or 9 in each of the two treatment groups, were included in each study. Dogs experimentally infected with the target parasite were dosed once on Day 0 with either placebo tablets or Simparica Trio™ tablets to provide minimum dosages of 1.2 mg/kg sarolaner, 24 µg/kg moxidectin and 5.0 mg/kg pyrantel (as pamoate salt). Timing of dosing relative to parasite inoculation allowed for efficacy to be evaluated primarily against the target parasite stage. Worm counts were conducted 7 or 8 days after treatments during necropsy. Efficacy was based on the number of worms recovered at necropsy compared to placebo control.

**Results:**

Based on geometric mean worm counts, efficacy of Simparica Trio™ was ≥ 98.4% against L_4_ larval stage of *A. caninum*, ≥ 99.8% against immature adult (L_5_) *A. caninum*, and 100% against adult *A. caninum* and adult *U. stenocephala.*

**Conclusions:**

These studies confirm the efficacy of a single oral dose of a novel, chewable tablet containing sarolaner, moxidectin and pyrantel (Simparica Trio™) against L_4_ larval and immature adult (L_5_) *A. caninum*, and adult *A. caninum* and *U. stenocephala* infections in dogs.

## Background

Ancylostomatids are nematode parasites that anchor themselves for feeding on the lining of the hosts’ small intestine using their hook-like mouthparts and are thus commonly known as ‘hookworms’. Hookworms are present worldwide, and *Ancylostoma caninum*, *Ancylostoma braziliense*, *Ancylostoma ceylanicum* and *Uncinaria stenocephala* are the species that most commonly infect dogs [1]. In general, *A. caninum* is found in warmer areas, *U. stenocephala* in colder areas of temperate and subarctic regions, and *A. braziliense* and *A. ceylanicum* in sub-tropical and tropical regions [2]. However, the geographical distribution of each species overlaps and their boundaries are not well characterized [1]. For example, *U. stenocephala*, which is generally thought to exist only in colder regions was identified as the most prevalent nematode in a canine intestinal parasite survey conducted in Cordova (Spain), which is a sub-tropical region [3].

The clinical signs of intestinal hookworm infection in the dog are those associated with blood loss, which occurs as a result of the multiple lacerations caused by attachment and re-attachment of the hookworm to the lining of the small intestine [4]. Severity of the clinical signs is dependent upon the hookworm species, level of infection, and age of the dog. Light infections in an adult dog may lead only to mild enteritis, while heavy infections in a nursing puppy can lead to acute anemia, circulatory collapse and death. Juvenile hookworms are of special concern since they may cause severe signs in young puppies before infections can be detected by routine fecal examination [2].

Soil becomes contaminated with infective third-stage hookworm larvae that have hatched and developed from eggs excreted in the host’s feces. Infection most commonly occurs when the infective larvae penetrate the skin, although infection can also occur by ingestion of infective larvae [1]. In puppies, a major source of *A. caninum* infection is by lactogenic transmission. When infection occurs, a portion of the larvae undergo somatic migration and can persist in the tissues in an arrested state for years [2]. These arrested larvae are reactivated during pregnancy and migrate to the mammary glands where they are transferred to nursing puppies [2]. Reactivation by unknown mechanism in non-pregnant dogs is also reported [5]. Humans can also become infected with hookworms by contact with infective larvae in contaminated soil. Infections in humans most commonly cause a self-limiting local dermatitis known as cutaneous *larva migrans* (CLM), which results from migrating larvae burrowing through the upper dermis [2]. Development to the adult stage in humans has also been shown to occur with some species [1]. *Ancylostoma braziliense* is believed to be the primary causative agent for CLM, although it is not certain that this is the only species involved [2].

To prevent the clinical consequences of hookworm infections, and to reduce environmental contamination, it is recommended that dogs receive anthelmintic treatment every two weeks from birth through eight weeks of age, followed by year-round monthly treatment [6]. It is also recommended that treatment efficacy be evaluated by fecal examinations conducted 2–4 times in the first year of life, and 1–2 times each year thereafter.

The studies presented here evaluated the efficacy of a novel, oral tablet containing sarolaner, moxidectin and pyrantel (Simparica Trio™, Zoetis, Parsippany, NJ, USA), against induced larval (L_4_), immature adult (L_5_), and adult *A. caninum* and induced adult *U. stenocephala* infections in dogs.

## Methods

Eight negative-controlled, masked, randomized laboratory studies were conducted. Studies were conducted according to the World Association for the Advancement of Veterinary Parasitology (WAAVP) guidelines for evaluating the efficacy of anthelmintics for dogs and cats [7], the International Co-operation on Harmonisation of Technical Requirements for Registration of Veterinary Medicinal Products (VICH) GL7, “Efficacy of anthelmintics: General requirements” [8], and with VICH GL19 “Efficacy of anthelmintics: Specific recommendations for canines” [9]. Personnel involved in making assessments of efficacy or safety were masked to treatment assignments.

### Animals

Purpose-bred laboratory Beagle or mixed breed dogs confirmed to be in good health by veterinary examination at the time of enrollment were selected. The selected dogs had undergone an adequate washout period to ensure that no residual activity remained from any previously administered anthelmintic compounds. Dogs were not allowed to be dewormed within 20 days of inoculation and for any previous deworming, only a short-acting anthelmintic with activity mainly limited to the gastrointestinal tract (e.g. pyrantel) was allowed. The administration of macrocyclic lactones was not permitted.

Dogs ranged in age from 7 to 13 weeks at the time of experimental hookworm inoculation and from 2.5 to 11.3 kg body weight at the time of treatment. Dogs were group housed prior to treatment, and individually housed after treatment. Housing enclosures conformed to accepted animal welfare guidelines [10, 11]. Dogs were fed an appropriate maintenance ration of a commercial canine diet for the duration of the study. Water was available *ad libitum.* Dogs were observed for general health at least once daily throughout the studies.

### Design

Four studies evaluated efficacy against immature stages of *A. caninum*: Studies 1 and 2 evaluated efficacy against L_4_ larvae, and Studies 3 and 4 evaluated efficacy against immature adults (L_5_). In Study 3, dogs were co-infected with *Toxocara canis* and the methodology and results are reported in a separate publication [12]. Co-infection was not expected to impact efficacy and that was also confirmed by the results. Four studies evaluated efficacy against adult hookworms: Studies 5 and 6 evaluated efficacy against adult *A. caninum* and Studies 7 and 8 evaluated efficacy against adult *U. stenocephala.* Study designs are summarized in Tables 1 and 2.

**Table 1:** Efficacy of a single oral dose of a novel chewable tablet (Simparica Trio™) containing sarolaner, moxidectin, and pyrantel pamoate against induced L_4_ larval and immature adult (L_5_) *Ancylostoma caninum* infections in dogs

Study	Isolate origin	Stage at time of treatment	Day of inoculation^a^	Day of treatment	Day of worm recovery	Treatment group^b^	*n*	No. of infected dogs	Worm count range	Geometric mean worm count	Efficacy compared to placebo	
											% Efficacy	Test statistic
1	Europe	L_4_ larvae	− 7	0	7	Placebo	9	9	11–36	21.0	–	–
						Simparica Trio™	9	0	0	0	100	*t*_(7)_ = 21.62 *P* < 0.0001
2	USA	L_4_ larvae			8	Placebo	8	8	4–17	9.2	–	–
						Simparica Trio™	8	1	0–2	0.1	98.4	*t*_(7)_ = 12.76 *P* < 0.0001
3	USA	Immature adults (L_5_)	− 11	0	7	Placebo	8	8	110–260	209.9	–	–
						Simparica Trio™	8	3	0–3	0.5	99.8	*t*_(7)_ = 22.77 *P* < 0.0001
4	Europe	Immature adults (L_5_)				Placebo	8	8	23–41	29.6	–	–
						Simparica Trio™	8	0	0	0	100	*t*_(7)_ = 47.93 *P* < 0.0001

^a^Each dog was inoculated with 200 ± 50 L_3_ *A. caninum*

^b^Simparica Trio™ provided minimum dosages of 1.2 mg/kg sarolaner, 24 µg/kg moxidectin and 5 mg/kg pyrantel (as pamoate salt)

*Abbreviation*: n, number of animals per group

**Table 2:** Efficacy of a single oral dose of a novel chewable tablet (Simparica Trio™) containing sarolaner, moxidectin, and pyrantel pamoate against induced adult *Ancylostoma caninum* and adult *Uncinaria stenocephala* infections in dogs

Study	Species (isolate origin)	Stage at time of treatment	Day of inoculation^a^	Day of treatment	Day of worm recovery	Treatment group^b^	*n*	No. of infected dogs	Worm count range	Geometric mean worm count	Efficacy compared to placebo	
											% Efficacy	Test statistic
5	*A. caninum* (Europe)	Adult	− 33	0	7	Placebo	8	8	59–136	109.9	–	–
						Simparica Trio™	8	0	0	0.0	100%	*t*_(7)_ = 47.61 *P* < 0.0001
6	*A. caninum* (USA)	Adult	− 32			Placebo	8	8	9–46	22.7	–	–
						Simparica Trio™	8	0	0	0.0	100%	*t*_(7)_ = 20.55 *P* < 0.0001
7	*U. stenocephala* (Europe)	Adult	− 32	0	7	Placebo	8	8	246–545	390.1	–	–
						Simparica Trio™	8	0	0	0.0	100%	*t*_(7)_ = 68.90 *P* < 0.0001
8	*U. stenocephala* (Europe)	Adult				Placebo	8	8	253–935	537.8	–	–
						Simparica Trio™	8	0	0	0.0	100%	*t*_(7)_ = 46.56 *P* < 0.0001

^a^In studies 5 and 6, each dog was inoculated with 200 ± 50 L_3_ *A. caninum* and in Studies 7 and 8 each dog was inoculated with 1250 ± 50 L_3_ *U. stenocephala*

^b^Simparica Trio™ provided minimum dosages of 1.2 mg/kg sarolaner, 24 µg/kg moxidectin, and 5 mg/kg pyrantel (as pamoate salt)

*Abbreviation*: n, number of animals per group

### Experimentally induced hookworm infections

Hookworms used for experimental inoculation were obtained from naturally infected dogs within approximately one year before use in Studies 1–6, and within approximately 6 years before use in Studies 7 and 8. The isolates were maintained by inoculation of donor dogs at regular intervals. Efficacy against each stage of *A. caninum* was evaluated against an isolate collected in Europe (Romania) and an isolate collected in the USA. The origin of the hookworm isolates is provided in Tables 1 and 2. Inoculum size and timing between inoculation of dogs and dosing was set based on the known life-cycle of the parasite to develop into the target stage and according to established guidelines [9].

In the studies that evaluated efficacy against immature stages of *A. caninum*, dogs were inoculated orally with 200 ± 50 L_3_ *A. caninum* larvae either 7 days prior to treatment (to evaluate efficacy against L_4_ larvae) or 11 days prior to treatment [to evaluate efficacy against immature (L_5_) adults]. In the studies that evaluated efficacy against adult *A. caninum*, dogs were inoculated orally with 200 ± 50 L_3_ *A. caninum* larva 32 or 33 days prior to treatment. In the studies to evaluate efficacy against adult *U. stenocephala*, dogs were inoculated orally with 1250 ± 50 L_3_ *U. stenocephala* larvae 32 days prior to treatment. The larvae counts were conducted by enumerating the viable larvae in representative aliquots of the larvae cultures used for inoculation using a microscope. Viability was confirmed by the motility of the larvae.

The total dose of infective L_3_ *A. caninum* or *U. stenocephala* inoculum for each dog was divided into two approximately equal doses which were administered approximately 4 h apart. Feed was withheld overnight prior to inoculation, and half of the dogs’ total daily feed ration was provided approximately 30 min after each inoculum administration. To alleviate emesis commonly caused by experimental hookworm infection [6], all dogs received an anti-emetic [Cerenia^®^ (maropitant citrate), Zoetis, Parsippany, USA] at the recommended label dose approximately 1 hour before the first inoculation.

### Randomization and treatment

Dogs were allocated randomly to treatment and pen according to a randomized complete block design. For the pre-patent L_4_ larvae and immature adult L_5_ *A. caninum* studies, block was based on pre-treatment body weight, and for the adult *A. caninum* and adult *U. stenocephala* studies block was based on pre-treatment fecal egg counts. Pre-treatment quantitative fecal egg counts were performed using a centrifugation-flotation technique [13] and the mean counts ranged from 182 to 1923 for *A. caninum* and from 628 to 1973 for *U. stenocephala.* For studies in which dogs were housed in multiple rooms (Studies 1, 4 and 5), dogs were grouped into blocks, randomized to treatment groups within block, then the blocks were randomly assigned to rooms and dogs randomly assigned to pens within room so that dogs within a block were housed in neighboring pens in the same room.

On Day 0, dogs were dosed orally with either placebo or Simparica Trio™ tablets. Each dog received one to three tablets of Simparica Trio™ to provide the minimum recommended dosages of 1.2 mg/kg sarolaner, 24 µg/kg moxidectin and 5 mg/kg pyrantel (as pamoate salt) or the equivalent number of placebo tablets. Body weights obtained within 4 days prior to dosing were used for dose calculation. Placebo and active tablet presentations were similar in appearance to maintain masking. Food was withheld overnight prior to treatment administration and was not offered again until approximately 4 h after treatment administration. All doses were administered by hand pilling to ensure accurate dosing. Each dog was observed for several minutes after dosing for evidence that the dose was swallowed.

### Necropsy and worm recovery

After food was withheld for approximately 15 h, dogs were humanely euthanized with phenobarbital sodium (intravenously, at the label dose) and necropsies were performed in a predetermined random order. After euthanasia the entire gastrointestinal tract from distal esophagus to rectum was removed, split longitudinally, and the mucosal surface was scraped twice to remove attached hookworms. For the L_4_ larval and L_5_ immature adult worm studies, the gastrointestinal contents and the scrapings were washed over a sieve with an aperture size of 38 µm. The scraped stomach and small intestine were then soaked in 0.9% saline solution and incubated at approximately 32.0–38.0 °C for approximately 2–4 h. After incubation, the soaked tissue was stripped twice to remove any released worms and the stripped material was washed over the sieve. For the adult worm studies, the stomach contents and the small intestine scrapings were washed over a sieve with an aperture size of 150 µm, and the large intestine contents washed over a sieve with a maximum aperture size of 300 µm. The contents of the sieves were rinsed, preserved in formalin and examined under magnification to identify and count recovered worms.

### Statistical analysis

The experimental unit was the individual dog and the efficacy endpoint was the total worm count at necropsy. Worm counts were transformed by the log_e_ (count + 1) transformation prior to analysis in order to stabilize the variance and normalize the data. Transformed counts were analyzed using a general mixed linear model (SAS 9.3 or 9.4, Cary NC) that included the fixed effect of treatment, and the random effects of block and error. In Studies 1, 4 and 5, the random effects included room, block within room, and error to account for housing in multiple rooms. Testing was two-sided at the significance level α = 0.05.

Percent efficacy relative to placebo was calculated using geometric means (back-transformed least square means) based on the formula [(C − T)/C] × 100, where C is the mean total worm count for the placebo group and T is the mean total worm count for the treated group.

## Results

There were no mortalities and no treatment-related adverse reactions in any study. Efficacy results against immature *A. caninum* are summarized in Table 1, and efficacy results against adult *A. caninum* and adult *U. stenocephala* in Table 2.

### L_4_ larval *A. caninum*

In both studies, 4–36 worms were recovered from each placebo-treated dog, confirming that the infection levels were adequate for determination of efficacy against L_4_ larval *A. caninum.* No worms were recovered from any of the Simparica Trio™-treated dogs in Study 1, and only 2 worms were recovered from a single Simparica Trio™-treated dog in Study 2. Geometric mean worm counts for the placebo groups in Studies 1 and 2 were 21.0 and 9.2, respectively, and for the Simparica Trio™ groups were 0 and 0.1, respectively. Mean worm counts in the Simparica Trio™ groups were significantly lower (12.76 ≤ *t*_(7)_ ≤ 21.62, *P* < 0.0001) than those for placebo and resulted in efficacies against L_4_ larval *A. caninum* of 100% in Study 1 and 98.4% in Study 2.

### Immature adult (L_5_) *A. caninum*

In both studies, 23–260 worms were recovered from each placebo-treated dog, confirming that the infection levels were adequate for determination of efficacy against immature adult (L_5_) *A. caninum.* It is noted that in some dogs more than 250 worms were recovered during necropsy, while dogs were inoculated with 200 ± 50 L_3_ *A. caninum* larvae. This discrepancy may be due to some larvae not showing any movement during their enumeration when preparing the inoculum. Therefore, these larvae could had been classified as non-viable and disregarded for the counts.

No worms were recovered from any of the Simparica Trio™-treated dogs in Study 4, and 1–3 worms were recovered from three Simparica Trio™-treated dogs in Study 3. Geometric mean worm counts for the placebo groups in Studies 3 and 4 were 209.9 and 29.6, respectively, and for the Simparica Trio™ groups were 0.5 and 0, respectively. Mean worm counts in the Simparica Trio™ groups were significantly lower (22.77 ≤ *t*_(7)_ ≤ 47.93, *P* < 0.0001) than those for placebo and resulted in efficacies against immature adult (L_5_) *A. caninum* of 99.8% in Study 3 and 100% in Study 4.

### Adult *A. caninum*

In both studies, 9–136 worms were recovered from each placebo-treated dog, confirming that the infection levels were adequate for determination of efficacy against adult *A. caninum.* No worms were recovered from any of the Simparica Trio™-treated dogs in either study. Geometric mean worm counts for the placebo groups in Studies 5 and 6 were 109.9 and 22.7, respectively. Mean worm counts in the Simparica Trio™ groups were significantly lower (20.55 ≤ *t*_(7)_ ≤ 47.61, *P* < 0.0001) than those for placebo and resulted in 100% efficacy against adult *A. caninum* in both studies.

### Adult *U. stenocephala*

In both studies, 246–935 worms were recovered from each placebo-treated dog, confirming that the infection levels were adequate for determination of efficacy against adult *U. stenocephala.* Geometric mean worm counts for the placebo groups in Studies 7 and 8 were 390.1 and 537.8, respectively. No worms were recovered from any of the Simparica Trio™-treated dogs in either study. Mean worm counts in the Simparica Trio™ groups were significantly lower (46.56 ≤ *t*_(7)_ ≤ 68.90, *P* < 0.0001) than those for placebo and resulted in 100% efficacy against adult *U. stenocephala* in both studies.

## Discussion

Dogs of all ages are at risk for infection with hookworms year-round for their entire lives. Treatment of puppies is critical to prevent potentially life-threatening clinical disease, and treatment of both puppies and adult dogs is essential to reduce environmental contamination thus reducing the risk of infection for dogs and humans.

In the studies presented here, a single oral dose of Simparica Trio™ provided ≥ 98.4% efficacy against L_4_ larval stage of *A. caninum*, ≥ 99.8% against immature adult (L_5_) *A. caninum*, and 100% efficacy against adult *A. caninum* and adult *U. stenocephala.* The efficacy of Simparica Trio™ was similarly high in all studies, including against the two different isolates of each species used that were collected from geographically distinct regions (USA and Europe), confirming similar susceptibility of the isolates against the active ingredients.

Both immature and adult *A. caninum* are voracious blood feeders, which can lead to significant clinical disease, including death due to blood-loss in young dogs. The efficacy provided by Simparica Trio™ against both immature and adult *A. caninum* will ensure a significant clinical benefit by eliminating gastrointestinal infections as early as possible. Efficacy against these immature gastrointestinal stages should also significantly reduce or even eliminate fecal egg shedding because female worms are killed before they can mature into egg-laying adults. Simparica Trio™ may be administered to dogs from 8 weeks of age and 1.25 kg body weight, ensuring that small puppies may also be safely dosed.

These laboratory results have been confirmed against natural hookworm infections under field conditions. In a European field study conducted at 45 veterinary clinics located in Germany, Hungary and Portugal, fecal egg counts were reduced by 99.0% in dogs naturally infected with *A. caninum*, and by 99.7% in dogs naturally infected with *U. stenocephala* 7 days after a single dose of Simparica Trio™ [14]. In a field study conducted at 18 sites located in different geographical regions of the USA, fecal egg counts were reduced by 98.6% in dogs naturally infected with *A. caninum* after a single dose of Simparica Trio™ [14]. In these European and USA field studies, Simparica Trio™ was also shown to be ≥ 99.0% effective against natural *T. canis* infections.

A variety of anthelmintic products are available for the treatment of *A. caninum* infections in dogs, and for many of these products the active ingredient is a macrocyclic lactone (e.g. milbemycin oxime, moxidectin) or a tetrahydropyrimidine (e.g. pyrantel). While most of these active ingredients also show efficacy against *U. stenocephala*, milbemycin oxime is an exception that does not achieve sufficient efficacy at the commonly approved minimal 0.5 mg/kg dose [15, 16]. This differentiation may be important in certain geographical regions where dogs have been found to be primarily infected with *U. stenocephala*, e.g. in Spain, UK, Ireland and Greece [3, 17–19]. Monthly administered products that include a macrocyclic lactone are particularly beneficial in that they not only provide control of most important intestinal parasitic nematodes of dogs but also provide control of the clinically important cardiopulmonary nematodes [2]. Simparica Trio™, which contains the macrocyclic lactone moxidectin in combination with sarolaner and pyrantel, has been shown to be effective in the prevention of both *Dirofilaria immitis* and *Angiostrongylus vasorum* [20, 21].

In addition to internal parasites, dogs are also at risk for infestation with fleas and ticks, which can have deleterious effects on their hosts including the transmission of disease agents to both dogs and humans; therefore, regular treatment of dogs at risk for these parasites is recommended [22–25]. The efficacy of Simparica Trio™ against flea and tick infestations both under field and laboratory conditions has been demonstrated [26–30].

The efficacy provided by the novel combination of sarolaner, moxidectin and pyrantel in Simparica Trio™ will be of benefit to veterinarians and pet owners by allowing for the monthly treatment of most of the internal and external parasites that commonly infect dogs in a single oral tablet.

## Conclusions

These studies confirm the efficacy of a single oral dose of a novel, chewable tablet containing sarolaner, moxidectin and pyrantel (Simparica Trio™) against immature and adult stages of *A. caninum,* and adult *U. stenocephala* infections in dogs.

## Data Availability

Data upon which the conclusions are based are provided within the article.

## Abbreviation

CLM: cutaneous *larva migrans*
